# Brazilian owners perception of the body condition score of dogs and cats

**DOI:** 10.1186/s12917-020-02679-8

**Published:** 2020-11-27

**Authors:** Fabio Alves Teixeira, Mariana Ramos Queiroz, Patrícia Massae Oba, Rodrigo Fernando Gomes Olivindo, Mariane Ceschin Ernandes, Caio Nogueira Duarte, Mariana Fragoso Rentas, Marcio Antonio Brunetto

**Affiliations:** 1 Department of Animal Nutrition and Production, School of Veterinary Medicine and Animal Science, University of São Paulo, Av. Duque de Caxias Norte, 255, Pirassununga, SP São Paulo, Brazil; 2 Department of Animal Sciences , University of Illinois at Urbana-Champaign , Illinois Urbana, USA

**Keywords:** canine, feline, obesity, nutritional assessment, body composition, nutritional tool

## Abstract

**Background:**

The knowledge of how owners view the body condition of their animals is an important factor for the success of the prevention/treatment of obesity and the engagement/adherence to nutritional interventions, which are fundamental to improve the animal prognosis. For this reason, the objective of this study was to evaluate the perception of the owners regarding the body condition score of their animals, compare the perception between cat vs. dog owners, and owners from the countryside vs. metropolitan region of São Paulo State in Brazil.

**Results:**

601 dogs and 110 cats were included in this study. There was no significant difference in body condition score assigned by owners by species. Owners of dogs and cats classified by the veterinarian as ideal, overweight, and obese who disagree with body condition score assigned by veterinarian mainly underestimate the body condition score of their animals, while lean dogs’ owners overestimate it. Countryside dog owners had a higher rate of disagreement with the veterinarian and more often underestimate the body condition score than owners from the metropolitan region. The owners of lean cats have the same judgments with veterinarians.

**Conclusions:**

Owners of dogs and cats have difficulty assessing the body condition score, especially owners from countryside.

## Background

The body condition score (BCS) is an important tool to determine the nutritional status of dogs and cats in clinical cases [1, 2]. Nowadays, obesity is the most commonly diagnosed nutritional disorder in cats and dogs and its prevalence is increasing in the last few years [3, 4]. BCS is the primary diagnostic tool for obesity [5]. Helping pet owners identify when their animals are getting overweight is essential to prevent obesity and avoid the consequences that this disorder can generate such as insulin resistance; diabetes mellitus; higher levels of inflammatory cytokines; hyperlipidemia; possible relation to cardiac, respiratory, skin, pancreatic and renal disease; hepatic lipidosis; osteoarthritis; negative impact on the quality of life and life span [6–16].

Besides, tracking the BCS assists in the monitoring of cardiac, renal, oncological, and endocrine diseases; as well as to determine the optimal time to initiate the intensive nutritional support for the hospitalized small animal patient [8, 17–23]. Furthermore, a stable BCS is associated with shorter hospitalization time and a higher hospital discharge rate, which is directly related to fewer costs to the owner [24–27].

The validated BCS for cats [28] and dogs [29] is a subjective semi-quantitative method, ranging from very thin (BSC = 1) to severely obese (BSC = 9). It involves an assessment of the visual and palpable characteristics of body fat at different body parts [1]. Studies conducted in other countries have shown that the owners tend to underestimate the BCS of their animals, especially obese animals [30–33]. In Brazil, studies only focus on the evaluation of overweight dogs were performed [34, 35]. For cats, high to moderate agreement between veterinary and owners perception of BCS, but among owners who incorrectly estimated the BCS of their cats, there is more underestimate, as found in the literature [36–40]. Only one study was found about Brazilian cat owners’ misperception of cat’s BCS, and it was conducted in a Brazilian countryside region [37]. Thus, there is no information regarding the perception of Brazilian owners regarding the BCS of their dogs and cats, as well as there is no comparison between the perceptions of dogs vs. cats owners.

Therefore, this study aimed to compare the perception of dogs and cats owners regarding the BCS of their animals; additionally, among owners who disagree with the BCS assigned by the veterinarian, if the propensity of the owners is to underestimate or overestimate the BCS of their animal; and to compare whether the BCS perception of dog and cat owners change according to them where they live (metropolitan region vs. countryside of São Paulo State, Brazil).

## Results

The study included 601 dogs and 110 cats. The distribution of animals by species, location, sex, most prevalent breeds and age is shown in Table 1. At metropolitan region, 39 canine breeds were included at study: Mongrel (23.9%), Poodle (16.4%), Labrador Retriever (10.3%), Spaniel Cocker (4.9%), Dachshund (4.9%), Yorkshire Terrier (4.2%), Lhasa Apso (3.4%), Pinscher (3.4%), Schnauzer (2.9%), Maltese (2.7%), Shih Tzu (2.4%), Beagle (2.1%), Bichon Frise (2.1%), Pomeranian (2.1%), Golden Retriever (1.9%), Pug (1.9%), Pit Bull (1.3%), English Bulldog (1.1%), Boxer (0.8%), Sharpei (0.8%), Akita (0.5%), Brazilian Terrier (0.5%), Germany Shepard (0.5%), Weimaraner (0.5%), Belgian Malinois (0.3%), Bernese (0.3%), Brazilian Mastiff (0.3%), Border Collie (0.3%), Bull Terrier (0.3%), Chow Chow (0.3%), French Mastiff (0.3%), English Setter (0.3%), Germany Pointer (0.3%), Mastiff (0.3%), Neapolitan Mastiff (0.3%), Rhodesian (0.3%), Rottweiler (0.3%), Scottish Terrier (0.3%) and West Highland White Terrier (0.3%). The feline breeds were: Mongrel (82.7%), Siamese (11.1%), Persian (5.0%) and Abyssinian (1.2%). At countryside, the canine breeds were: Mongrel (50.0%), Poodle (4.9%), Dachshund (4.5%), Shih Tzu (4.5%), Yorkshire Terrier (4.0%), Lhasa Apso (3.6%), American Pit Bull (3.2%), Pinscher (3.2%), Border Collie (2.7%), Golden Retriever (2.7%), Labrador Retriever (2.7%), Beagle (1.8%), Pug (1.8%), Rottweiler (1.4%), Akita (0.9%), Boxer (0.9%), Brazilian Terrier (0.9%), Cocker (0.9%), Maltese (0.9%), Schnauzer (0.9%), Australian Cattle Dog (0.4%), Bernese (0.4%), Bichon Frise (0.4%), Chihuahua (0.4%), Chow Chow (0.4%), Germany Shepherd (0.4%), Pomeranian (0.4%), Siberian Husky (0.4%) and West Highland White Terrier (0.4%). At this region, feline breeds were Mongrel (96.6%) and Persian (3.4%).

The proportions of underestimation, agreement, and overestimation of BCS by owners of dogs and cats, separated by owners residing in the metropolitan and countryside region, and the degree of agreement by the linear weighted kappa test are available in Table 2.

**Table 1:** Description of the metropolitan and countryside region animals included in the study

	Metropolitan		Countryside	
	Dogs	Cats	Dogs	Cats
Number of animals	377	81	224	29
Sex (M%/F%)	43.2/56.8	40.7/59.3	36.6/63.4	24.1/75.9
Age (years; average ± sd)	9.4 ± 3.7	9.5 ± 4.5	6.9 ± 4.3	5.4 ± 4.2
Breeds (n)	39	4	29	2
More prevalent breeds (%)	Mongrel (23.9)	Mongrel (82.7)	Mongrel (50.0)	Mongrel (96.6)
	Poodle (16.5)	Siamese (11.1)	Poodle (4.9)	Persian (3.4)
	Labrador (10.3)	Persian (4.9)	Dachshund (4.5)	-

*n *number of breeds; *M% *percentage of males; *F% *percentage of females; *sd* standard deviation

**Fig. 1:**
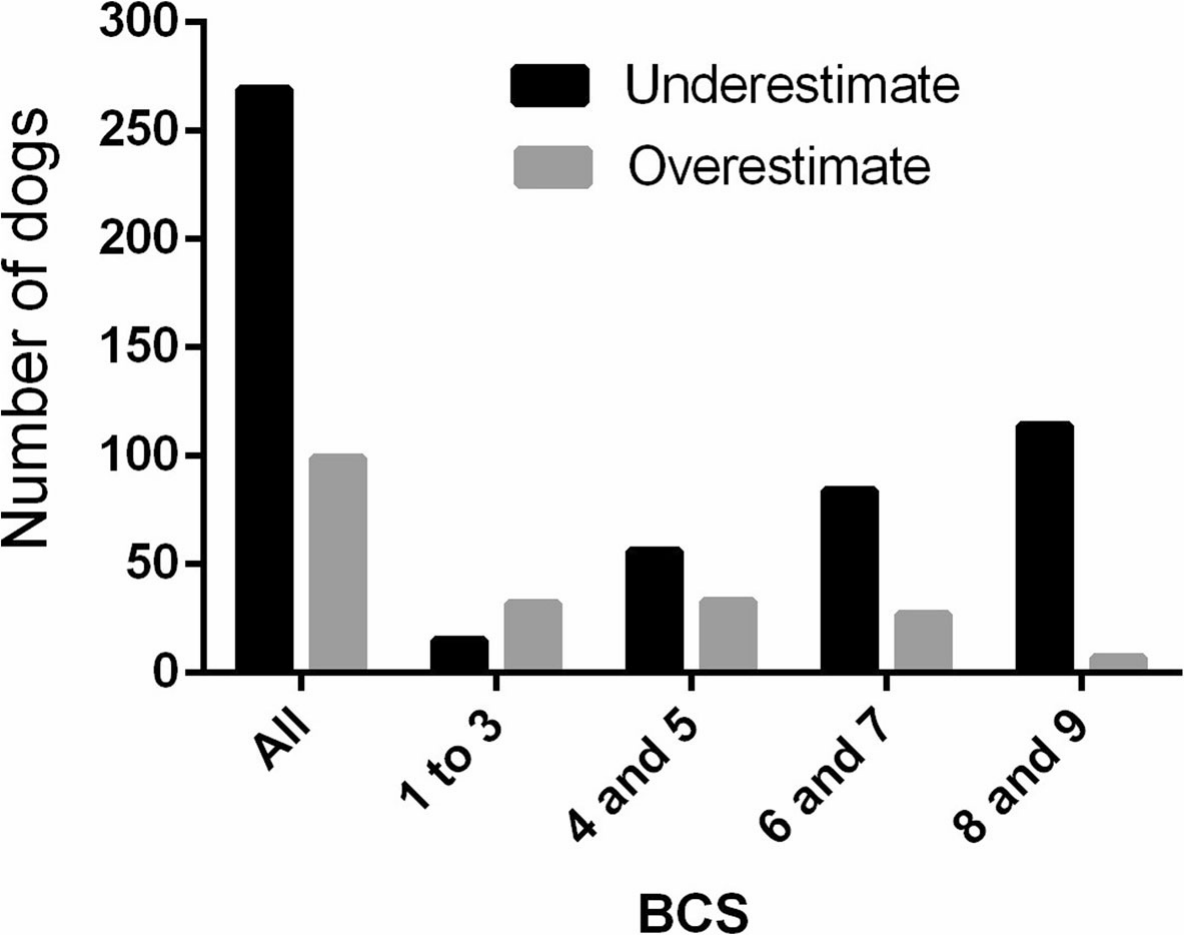
Evaluation of the propensity of the owners, who disagreed with the veterinarian, in underestimating or overestimating the body condition score (BCS) of dogs

**Fig. 2:**
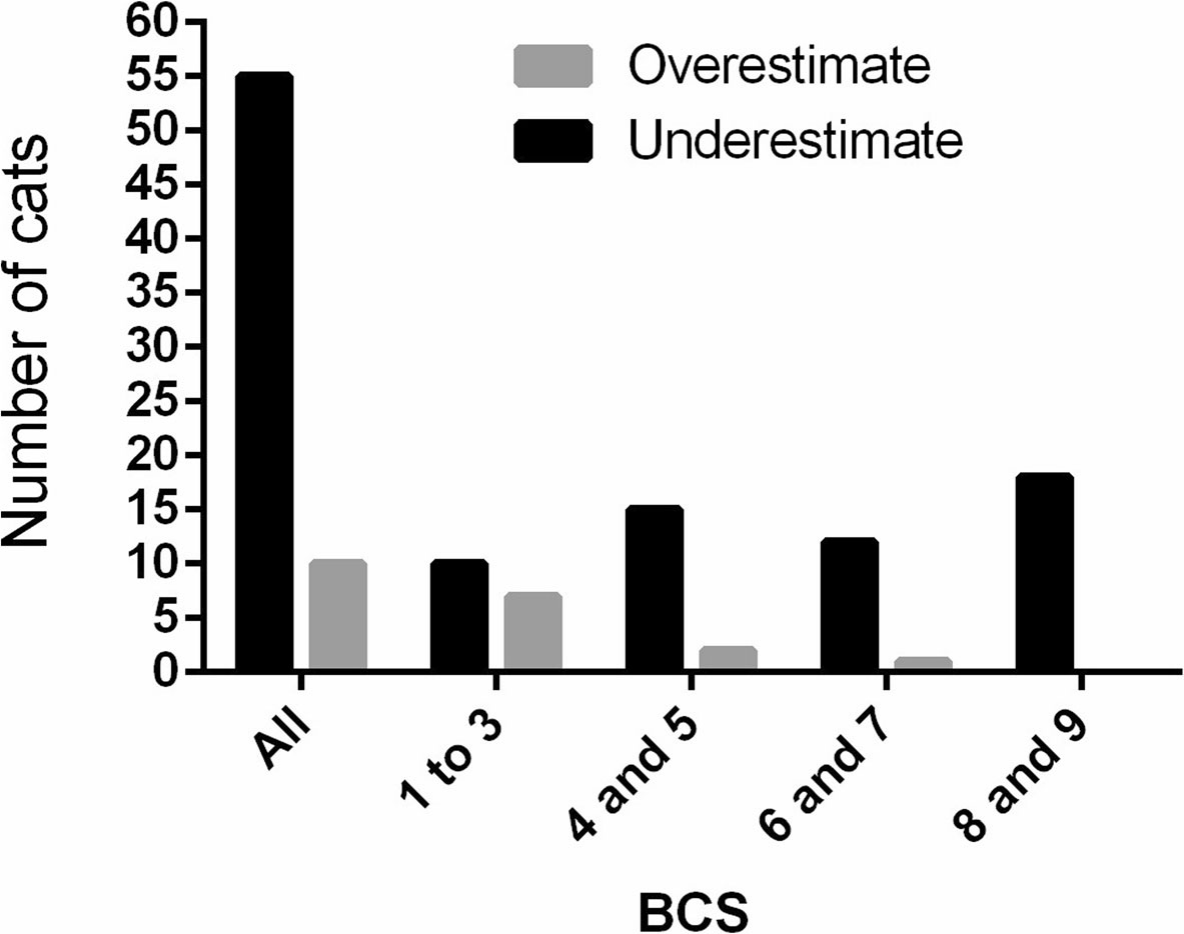
Evaluation of the propensity of the owners, who disagreed with the veterinarian, in underestimating or overestimating the body condition score (BCS) of cats

When data were analyzed considering all BCS ranges (Table 2), in general, and in the metropolitan region the dogs and cats owners showed a high degree of agreement between the body condition score classified by owners (BCSo) vs. body condition score classified by veterinarian (BCSv) (κ = 0.64 to 0.69; *p* < 0.01) and in the countryside the agreement was considered as moderate (κ = 0.54 and 0.58; *p *< 0.01). However, when evaluating the distribution of disagreement with the BCSv it is possible to notice that, regardless of the region, the dog (73.1% vs. 26.9%; *p* < 0.01) and cats (84.6% vs. 15.4%; *p* < 0.01) owners, who disagreed with BCSv, more frequently underestimate the BCS of their animals than overestimate it (Figs. 1 and 2).

Furthermore, comparing dogs vs. cats owners, there was no difference in the proportions of underestimation, agreement, or overestimation (*p* > 0.05). Among the regions, it was possible to notice that the owners from the metropolitan region are more likely to agree with the veterinarian compared to the countryside region (44.0% vs. 29.9%; *p* < 0.01), they are unlikely to underestimate (35.0% vs. 61.2%; *p* < 0.01) and likely to overestimate the body condition of their dogs (21.0% vs. 8.9%; *p* < 0.01). Whereas, metropolitan cats owners had a lower underestimate ratio of the BCS compared to the residents of countryside region (43.2% vs. 69.0%; *p* = 0.03), without different (*p* > 0.05) for agreement and overestimation.

However, when these results are analyzed by the BCS group, the degree of agreement varied from low to moderate: for lean dogs, the agreement was reasonable in the metropolitan area (κ = 0.31; *p* < 0.01) and moderate in the countryside (κ = 0.42; *p* < 0.01); dogs with ideal BCS, the agreement was reasonable in the metropolitan area (κ = 0.24; *p* < 0.01) and without significance to demonstrate the degree of agreement in the countryside (*p* = 0.11); and for both overweight and obese dogs the agreement was low regardless of the region (κ = 0.07 to 0.18; *p* < 0 0.01).

For cats, when the total number of animals per BCS group was analyzed, it was possible to state that the degree of agreement was moderate for lean animals (κ = 0.45; *p* < 0.01) and low for obese animals (κ = 0.17; *p* = 0.01). Among the owners of the metropolitan region the degree of agreement was moderate (κ = 0.42; *p* < 0.01) between BCSo and BCSv in the group of lean cats. For the remaining BCS groups, the degree of agreement could not be determined due to non-significance in the linear weighted kappa test (*p* > 0.05), probably due to the low number of cats in the present study.

When BCS perception among dog and cat owners was compared, there was only a difference in the group of animals with ideal BCS in the metropolitan region (*p* < 0.01), in which cats owners seem to more frequently underestimate the BCS of their animal than dogs owners (63.2 vs. 27.8%).

Between the metropolitan region and countryside region, when the data are analyzed separated by the BCS groups, there was no difference between the perception of cats owners in the proportion of underestimation, agreement, and overestimation (p > 0.05). However, it was observed that dog owners in the countryside region had a higher ratio of underestimate the BCS of dogs in ideal condition (52.5% vs. 27.8%; *p* < 0.01), overweight (67.6% vs. 31.5%; *p* < 0.01) and obesity (82.8% vs. 51.1%; *p* < 0.01); and had a lower agreement ratio with the veterinarian when dogs are overweight (28.4% vs. 46.3%; *p* = 0.02) and obese (17.2% vs. 41.9%; *p* < 0.01); and consequently had a lower overestimate level of the BCS of overweight dogs (4.1% vs. 22.2%; *p* < 0.01).

When comparing underestimation and overestimation (Table 2), it was observed that the dogs owners that do not agree with the BCSv are likely to overestimation of the BCS of lean dogs (68.1% vs. 31.9%; p < 0.01), and underestimation of the BCS of dogs in ideal condition (62.9% vs. 37.1%; *p* < 0.01), overweight (75.7% vs. 24.3%; *p* < 0, 01) and obese (94.2% vs. 5.8%; *p* < 0.01). There is also a greater susceptibility to underestimation of BCS among owners of obese felines (100.0% vs. 0.0%; *p* < 0.01), overweight (92.3% vs. 1.7%; *p* < 0.01) and ideal condition (88.2% vs. 11.8%; *p* < 0.01). Furthermore, among the lean cats there was no difference in the proportion of underestimation and overestimation (58.8% vs. 41.2%; *p* = 0.49).

**Table 2:** The degree of agreement and comparison between underestimation, agreement, and overestimation proportions of BCS attributed by dog and cat owners in the metropolitan region and countryside region considering all animals and separated by BCS range

	All regions		Metropolitan region		Countryside region	
	Dogs	Cats	Dogs	Cats	Dogs	Cats
ALL BODY CONDITIONS SCORES (BCS 1 to 9)						
n	601	110	377	81	224	29
Underestimation	44.8%	50.0%	35.0%	43.2%	61.2%	69.0%
Agreement	38.8%	40.9%	44.0%	44.4%	29.9%	31.0%
Overestimation	16.5%	9.1%	21.0%	12.4%	8.9%	0.0%
Kappa	0.64	0.69	0.69	0.69	0.54	0.58
p*	< 0.01	< 0.01	< 0.01	< 0.01	< 0.01	< 0.01
Degree of agreement	High	High	High	High	Moderate	Moderate
LEAN (BCS 1 to 3)						
n	89 (14.8%)	39 (35.5%)	62 (16.4%)	37 (45.7%)	27 (12.1%)	2 (6.9%)
n (BCSo)	100 (16.6%)	49 (44.5%)	67 (17.8%)	45 (55.4%)	33 (14.7%)	4 (13.9%)
Underestimation	16.9%	25.6%	19.4%	27.0%	11.1%	0.0%
Agreement	47.2%	56.4%	43.6%	54.1%	55.6%	100.0%
Overestimation	36.0%	17.9%	37.1%	18.9%	33.3%	0.0%
Kappa	0.34	0.45	0.31	0.42	0.42	-
p*	< 0.01	< 0.01	< 0.01	< 0.01	< 0.01	-
Degree of agreement	Reasonable	Moderate	Reasonable	Moderate	Moderate	-
IDEAL (BCS 4 and 5)						
n	149 (24.8%)	26 23.6%)	90 (23.9%)	19 (23.5%)	59 (26.3%)	7 (24.1%)
n (BCSo)	177 (29.5%)	28 (25.5%)	90 (23.9%)	16 (19.8%)	87 (38.8%)	12 (41.7%)
Underestimation	37.6%	57.7%	27.8%	63.2%	52.5%	42.9%
Agreement	40.3%	34.6%	44.4%	26.3%	33.9%	57.1%
Overestimation	22.2%	7.7%	27.8%	10.5%	13.6%	0.0%
Kappa	0.20	0.10	0.24	0.03	0.11	0.36
p*	< 0.01	0.19	< 0.01	0.72	0.11	0.15
Degree of agreement	Low	-	Reasonable	-	-	-
OVERWEIGHT (BCS 6 and 7)						
n	182 (30.3%)	17 (15.5%)	108 (28.6%)	8 (9.9%)	74 (33.0%)	9 (33.3%)
n (BCSo)	225 (37.4%)	18 (16.4%)	139 (36.9%)	9 (11.1%)	86 (38.4%)	9 (33.3%)
Underestimation	46.2%	70.6%	31.5%	50.0%	67.6%	88.9%
Agreement	39.0%	23.5%	46.3%	37.5%	28.4%	11.1%
Overestimation	14.8%	5.9%	22.2%	12.5%	4.1%	0.0%
Kappa	0.18	0.13	0.20	0.11	0.10	0.07
p*	< 0.01	0.05	< 0.01	0.29	0.01	0.27
Degree of agreement	Low	-	Low	-	Low	-
OBESE (BCS 8 and 9)						
n	181 (30.1%)	28 (25.5%)	117 (31.0%)	17 (21.0%)	64 (28.6%)	11 (37.9%)
n (BCSo)	99 (16.5%)	15 (13.6%)	81 (21.5%)	11 (13.6%)	18 (8.0%)	4 (11.1%)
Underestimation	63.0%	64.3%	51.1%	52.9%	82.8%	81.8%
Agreement	33.2%	35.7%	41.9%	47.1%	17.2%	18.2%
Overestimation	3.9%	0.0%	6.0%	0.0%	0.0%	0.0%
Kappa	0.14	0.17	0.18	0.18	0.07	0.10
p*	< 0.01	0.01	< 0.01	0.05	< 0.01	0.17
Degree of agreement	Low	Low	Low	-	Low	-

*n* number of animals; *BCSo* body condition score classified by owners; ^*^Assessment of the degree of agreement between the BCS assigned by the owner and the veterinarian according to Kappa Weighted Test. The result was considered significant when *p* < 0.05

## Discussion

Considering the subjectivity of the BCS used [28, 29] and the low frequency of use of this method by veterinarians [41], the study was conducted by the veterinary clinical nutrition team. These veterinarians have extensive experience in the evaluation of the body condition of dogs and cats, which allowed BCSv to be used as a gold standard since this method has a good correlation with more objective alternatives such as dual-energy x-ray absorptiometry for establishing the body composition when executed by experienced professionals [42]. Although it has been shown that the use of pictures does not improve the perception of the dog owners regarding the actual body condition of their animals [31, 32, 35], this study chose to use an illustrative chart of the BCS of dogs and cats, with a nine-point scale, to explain to the owners each body condition point, since its use seems to be a modifying factor of BCS attribution by cat owners [40] and the dog owners consider that the pictures help in the assignment of BCS [31]. The results observed in the present study show that Brazilian pet owners have difficulty in assessing the body condition of their animals, 61.2% of dog owners and 59.1% of cat owners disagree with BCSv. These results are close to those observed in the literature, where it was previously reported a range of 44.1–72% in the proportion of disagreement by dog owners [31–33, 35] and 47.3% disagreement by cats owners [40].

Moreover, there was a similarity between the previous studies and the present study in the distribution of the disagreement between BCSv and BCSo. In this study, as in other publications [30, 31], the owners of lean dogs, when in disagreement with the veterinarian, overestimated the animal’s body condition, and owners of overweight and obese dogs underestimate the BCS. The fact that the dog owners classify the leans animals as being in healthier body condition was also observed by White et al. [33] and was associated with the owner’s optimism in seeing their animals recovering from some illness that led to weight loss. Furthermore, a similar result was found in a past study with cat owners [40], the owners of lean cats who disagree with the veterinarian, overestimated the BCS of their animal, while those in ideal condition, overweight, and obese underestimated it. The difference in the present study is that there was no difference between underestimation and overestimation among the owners of leans animals that disagreed with BCSv.

Specifically regarding the group of obese animals, in both species, there was an underestimation of the body condition by the majority of the owners, to the point that the dogs linearized kappa values decreased as the BCS increased, which resulted in a low degree of agreement in the dogs. Additionally, in the present study, 30.1% of the dogs and 25.5% of the cats were diagnosed with obesity by the veterinarians (BCS ≥ 8), while only 16.5% and 13.6% (respectively) of the owners agreed with the BCSv. Likewise, Singh et al. [32] reported that 79% of the dogs were racked as overweight by the veterinarians, whereas only 28% of the dogs were scored in this condition by the owners. A Brazilian study published by Aptekmann et al. [34], with a focus on overweight dogs, reported 52% of dogs with BCS ≥ 8, however, only 27% were obese in the owner’s opinion. Likewise, in another study conducted in Brazil by Carciofi et al. [35], selected only obese dogs, 52% of owners underestimated the BCS of their animals.

This distinction between the regions in the state of São Paulo may be explained by the theory that people in large urban centers treat their animals like family and consequently are more concerned with pet health. However, this study did not consider the characteristics of the owners as their body condition, educational level, social class, type of interaction with pets etc., factors that could interfere in the way they perceive the BCS of their dogs, as observed by Courcier et al. [30] and that would be interesting parameters to be used as predisposing to owners who erroneously perceive the animal’s body condition [44]. This shows that the results of this study should be analyzed with thoughtfulness, as they probably do not reflect the reality of the perception of the entire Brazilian population regarding the body condition of their animals.

With the humanization of pets, pet owner behaves like parents of children, and do not perceive the degree of obesity of the children [45, 46]. Just as in human medicine, in what efforts are made for humans to recognize the body condition of their children and their own, in veterinary medicine to know how the owners see the BCS of their animals is fundamental for new interventions to be established prevention and treatment of obesity and other nutritional disorders. Owners that view their animals as in lower BCS than they are is harmful because, some owners may try to increase their pet’s weight in pursuit of what they believe to be the proper body condition, and make it difficult for the veterinarian to establish a weight loss program. Likewise, the propensity to see their animal in an ideal body condition when the animal is lean can be a problem, making it difficult to establish intensive nutritional support to pets that do not ingest the adequate amount of nutrients and energy and the owners may not accept the veterinary intervention.

The nutritional assessment guide developed and endorsed by various veterinary entities recommends that the owners be educated by the veterinarian to recognize the differences between the BCS points [47, 48] since the success in the treatment of obesity is linked to the adherence of the owners to veterinary prescriptions, which depends on the awareness of that their animal is above [14] or below the ideal body condition and this can be harmful to the health of their animal [8, 49]. Thus, research with this focus on veterinary medicine should be encouraged. This study evidences the discrepancy between owners and veterinarians regarding the body condition of their animal. The consistency between the BCS of a veterinarian and the owners is a key factor for the success of a weight loss program and the implementation of intensive nutritional support.

Some research has already evaluated the perception of dogs and cats owners regarding the body condition of their pets. However, the innovative character of this study is the fact that data were established in Brazilian territory, different from the studies that developed their work with dogs in the United Kingdom [30, 31, 33] and cats in France [40] or worked in Brazil only with dogs in overweight and obese [34]. Furthermore, to the best of our knowledge, this is the first study that compared the perception of BCS by dogs and cats owners and found a difference in the perception between the owners of both species only when the data were analyzed separately by the cities and by BCS groups: dogs that lived in the metropolitan area in ideal body condition had the BCS more frequently underestimated by their owners than the cats that lived in the same area (63.2% vs. 27.8%, *p* = 0.01). This could be attributed to the fact that dogs owners who exercise with their pets could consider physical activity as an influencing factor and thus underestimate the weight of the animal, however, this study was not focused on this hypothesis and therefore this study was not designed to confirm this hypothesis in the present study.

Another limitation of this study was the lower number of owners of cats when compared to the number of owners of dogs and the possibility of biases by the type of convenience sampling: the owners interviewed were those attended by the veterinary clinical nutrition team, which may have generated some degree of improvement in the perception of their pet’s body condition because they have previously talked to other veterinarians about the weight of their animals. As stated by White et al. [33], a greater degree of agreement between the body condition scored by the veterinarian and by the owners is associated with a high percentage (75%) of owners having already discussed with their veterinarian about their dog’s weight. It is important to highlight that we did not have inclusion criteria for participation in the study since we had more overweight and obese dogs than cats for nutritional evaluation.

The present study has also emphasized the need to educate the public regarding companion animal obesity. In this respect, most of the owners interviewed had no prior knowledge of BCS and how to use it. This might be due to the fact that veterinarians do not commonly weigh dogs or estimate their body condition [42] therefore, they do not record the overweight status of pets [43]. Tackling owner misperception of body condition is arguably one way whereby veterinarians could improve owner awareness of a healthy weight and the importance to avoid obesity.

Moreover, the present study observed that the perception of dog owners is modified according to the region in which they reside: countryside owners see their dogs leaner than they really are and this may negatively impact the prevalence and treatment of pet obesity in these regions. As for the cat owners, this difference was not observed, but comparatively, French cat owners agree more with the veterinarian than Brazilian cat owners: 40.9% vs. 60% (Brazilian vs. French cat owners) [40].

## Conclusions

The results obtained here was no significant difference in the perception of BCS by owners of dogs and cats. There is a need to educate the public regarding companion animal obesity and malnutrition, considering the differences between pet owners in the metropolitan areas and the countryside, in addition to using the BCS as a tool to improve communication between veterinarians and pet owners, to make the owners accept and adherence the nutritional interventions necessary for the treatment of the animal.

## Methods

During the first appointment carried out by the veterinary clinical nutrition team between October 2013 and May 2018, all animals older than 1 year were enrolled in the study, regardless of diagnosis. Veterinarians explained to cat and dog owners how to assess the BCS using an illustration with a nine-point scale [28, 29] to exemplify the score. The illustrations chart translated into Brazilian Portuguese is made available in Brazil by the pet food company Nestlé Purina (Ribeirão Preto, SP, Brazil). The owners classified their animals BCS (BCSo) without interference from the veterinarian. The BCS assigned by the veterinarians (BCSv) was used as the standard to determine the degree of agreement between the BCSo and the accurate BCS. All veterinarians involved in this study were part of the same clinical nutrition team and underwent the same form of training prior to the BCS assessment. All animals remain with their owners during the appointment and returned to their homes after it.

Analyses were performed considering all data (regardless of the region where the appointments were held), the data from the metropolitan region (São Paulo city − 11,253,503 inhabitants and 7,387.96 inhabitants per km^2^ [50]) vs. the countryside region (Pirassununga city − 70,081 inhabitants and a demographic density of 96.38 inhabitants/km^2^ [50]) of São Paulo state, Brazil, and data from dogs and cats owners. BCS perceptions were evaluated using the nine-point scale [28, 29] or subdivided into four body condition groups: lean (1 to 3), ideal (4 and 5), overweight (6 and 7), and obese (8 and 9).

The statistical analyses were performed in R Core Team software (2016)^2^. The degree of concordance between BCSo and BCSv was assessed by the linear weighted Kappa (Kp) test that verifies if there is more agreement that would occur due to chance. According to the results of this test the degree of agreement is categorized as very low (κ < 0.00), low (0.00 to 0.20), reasonable (0.21 to 0.40), moderate (0.41 to 0.60), high (0.61 to 0.80) and almost perfect (0.81 to 1.00) [51]. A chi-square test of proportions equality was used to test if there was a difference between the perceptions of dog vs. cats owners, between owners from metropolitan vs. countryside areas of São Paulo state, and the likelihood of owners who disagreed with the BCSv is to underestimate or overestimate the BCS of their animal. For both tests, the results were considered significant when the p-value was less than 0.05.

## Data Availability

The datasets used and analysed during the current study are available from the corresponding author on reasonable request.

## Footnotes

2 R Core Team. R: A Language and Environment for Statistical Computing. R Foundation for StatisticalComputing. Vienna, Austria, 2016.
